# Ultrasound‐Responsive Polymeric Piezoelectric Nanoparticles for Remote Activation and Neuronal Differentiation of Human Neural Stem Cells

**DOI:** 10.1002/smsc.202400354

**Published:** 2024-11-27

**Authors:** Arianna Bargero, Matteo Battaglini, Tommaso Curiale, Alessio Carmignani, Margherita Montorsi, Massimiliano Labardi, Carlotta Pucci, Attilio Marino, Gianni Ciofani

**Affiliations:** ^1^ Istituto Italiano di Tecnologia Smart Bio‐Interfaces Viale Rinaldo Piaggio 34 56025 Pontedera Italy; ^2^ Department of Mechanical and Aerospace Engineering Politecnico di Torino Corso Duca degli Abruzzi 24 10129 Torino Italy; ^3^ The Biorobotics Institute Scuola Superiore Sant’Anna Viale Rinaldo Piaggio 34 56025 Pontedera Italy; ^4^ Sede Secondaria di Pisa CNR‐IPCF Largo Pontecorvo 3 56127 Pisa Italy

**Keywords:** neural differentiations, neural stem cells, piezoelectric nanoparticles, regenerative medicines, ultrasound‐responsive nanomaterials

## Abstract

The regenerative capacity of the central nervous system (CNS) is limited. Understanding and enhancing the mechanisms that induce neural differentiation of neural stem cells (NSCs) is crucial for advancing regenerative medicine; one significant challenge in this effort is the remote delivery of pro‐differentiation cues. In this framework, a nanotechnology‐based solution able to remotely trigger the differentiation of human NSCs (hNSCs) into neurons is proposed. The approach involves organic piezoelectric nanotransducers, which can be remotely activated by low‐intensity ultrasound (US) for local and noninvasive electrical stimulation. Highly biocompatible piezoelectric polymeric nanoparticles, when activated by US, demonstrate the ability to induce calcium influx, exit from the cell cycle, and neuronal differentiation in hNSCs, as evidenced by calcium imaging experiments and the expression analysis of the NeuN post‐mitotic neural marker; additionally, an increased outgrowth of the developing axons is observed. Gene expression analysis moreover suggests that the neural differentiation mechanism induced by piezoelectric stimulation acts by upregulating the calcium signaling‐sensitive NeuroD1 neural inducer and the Lamb1 marker, independently of the c‐Jun/c‐Fos pathway. Considering the high biocompatibility and the good piezoelectricity of the polymeric nanotransducers used in this work, it is believed that this “wireless” stimulation approach holds high potential in CNS regenerative medicine.

## Graphical abstract

A nanotechnology‐based solution using polymeric piezoelectric nanotransducers activated by low‐intensity ultrasound is proposed to remotely stimulate human neural stem cell differentiation. In the results, promising neuronal differentiation and axon outgrowth are shown, highlighting the potential of this approach for central nervous system regenerative medicine.

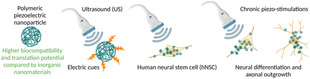

## 1 Introduction

The adult central nervous system (CNS) shows limited regeneration capability.^[^ ^1^, ^2^ ^]^ Although small niches of neural stem cells (NSCs) can compensate for the limited extent of neuronal loss, this mechanism fails in case of significant neuronal damage. One promising therapeutic strategy to support neuroregeneration is represented by the NSC transplant.^[^ ^3^, ^4^ ^]^ Exogenous NSCs, induced to differentiate in vitro or in vivo, may offer a solution for neuronal replacement. In addition, transplanted NSCs can induce neuroprotective and trophic effects.^[^ ^5^, ^6^ ^]^ Understanding the activation, differentiation, and integration mechanisms of NSCs and learning how to control them are crucial for effective therapy.^[^ ^7^, ^8^, ^9^ ^]^ These cells are known to be sensitive to various stimuli, including biochemical and physical stimuli (e.g., mechanical, electrical, magnetic, optical, thermal, topographical, etc.).^[^ ^10^, ^11^, ^12^, ^13^, ^14^, ^15^, ^16^ ^]^ The comprehension about how these stimuli affect them, individually or in combination, is essential to gain greater control over differentiation processes, and thus improve their effectiveness.^[^ ^9^ ^]^

Given that neurons are electrically active cells, electrical stimulation (ES) is a powerful tool to promote neuronal differentiation from NSCs. Within the CNS, innate electrical activity is critical in promoting tissue regeneration by attracting NSCs to the site of injury; conversely, exogenous electrical activity involves the artificial stimulation of action potentials that produce an electrical charge in the cell.^[^ ^12^, ^17^ ^]^ ES can be applied both in vivo and in vitro and can significantly enhance NSC proliferation or neuronal differentiation, depending on the stimulation parameters used, such as frequency, duration, voltage, and current.^[^ ^12^ ^]^ The biophysical changes initially occur at the cell surface and affect membrane protein functions, including enzyme activity, membrane receptor complexes, and ion transport channels, by altering charge distribution.^[^ ^12^ ^]^ ES generally falls into two categories: electric field (EF) stimulation and electromagnetic field (EMF) stimulation. EF is typically applied to biological tissues using electrodes and can be classified according to the current applied: direct (DCEFs), alternating, pulsed, and biphasic.^[^ ^12^ ^]^ Several studies have demonstrated the effectiveness of each of these stimulation techniques in enhancing neuronal differentiation.^[^ ^18^, ^19^, ^20^, ^21^ ^]^ Currently, the most widely used is DCEFs, which also promote neurite growth and rapid neural extension of induced neurons by regulating calcium ion levels. EMFs represent a noninvasive method that has garnered significant research attention for brain stimulation. Transcranial magnetic field stimulation (TMS), for example, promotes adult hippocampal neurogenesis and increases the number of newborn neurons in the granule cell layer of the dentate gyrus in vivo,^[^ ^22^ ^]^ while numerous studies report that low‐frequency and low‐intensity continuous magnetic field stimulation has a beneficial effect on NSC neurogenesis and proliferation.^[^ ^23^ ^]^

Compared to chemically induced differentiation, ES has the advantage of precise control of stimulation through on/off switching and selective stimulation regions, as cells exposed to ES can be easily selected based on electrode placement. It can also be easily manipulated by an external power source. The limitations are mainly related to the parameterization of the EF, which needs to be optimized according to the types of cells stimulated and the desired effect. Even small changes in the parameters can significantly affect the cell response.^[^ ^17^ ^]^ Currently, the most commonly used stimulation techniques include optogenetics, deep brain stimulation, transcranial direct current stimulation, and TMS. However, these techniques are associated with numerous problems, such as poor tissue permeability to visible light, the need for invasive electrodes that cause infection and gliosis, and low spatial resolution that hinders precise stimulus delivery.^[^ ^24^ ^]^ Therefore, one of the main challenges is to achieve precise ES with noninvasive techniques.

One possible solution is the use of piezoelectric materials,^[^ ^25^, ^26^ ^]^ applicable in wireless therapeutic approaches where mechanical energy (e.g., physical bending, ultrasound [US], water flow, mechanical stirring) is used to induce an electrical response (direct piezoelectric effect). The concept of remotely generating an electrical charge by combining piezoelectric nanoparticles and USs represents an emerging therapeutic approach^[^ ^27^ ^]^ presenting several advantages over other remote stimulation techniques. The use of US allows for effective tissue penetration (up to 5 cm) compared for example to light, making it optimal for wireless electrical stimulus delivery.^[^ ^27^, ^28^ ^]^ In addition, the electrical response is elicited each time mechanical stress is applied, allowing for periodic or continuous stimulation.

Among the piezopolymers, poly(vinylidene fluoride) (PVDF) and its copolymer poly(vinylidene fluoride‐trifluoroethylene) (P(VDF‐TrFE)) are among the few ones exhibiting a good level of piezoelectricity, with coefficient values comparable to some inorganic materials.^[^ ^29^ ^]^ A notable study by Hoop et al.^[^ ^30^ ^]^ explored the use of PVDF membranes as piezoelectric substrates for wireless neuronal differentiation, focusing on the differentiation of PC12 cells via US‐induced electrical charges on the membrane surface. They demonstrated that this approach could activate Ca^2+^ channels and promote neuritogenesis in a mechanism independent of growth factors like nerve growth factor (NGF). However, their system relied on PVDF membranes, which are not ideal for all applications, particularly where nanodispersions and noninvasive delivery systems are preferred. In contrast, our work explores the use of P(VDF–TrFE) nanoparticles (PNPs), which offer several advantages over PVDF membranes. The P(VDF–TrFE) copolymer moreover exhibits superior piezoelectric properties due to its enhanced crystallinity and dipolar alignment, making it a more efficient and flexible platform for inducing ES at the nanoscale.^[^ ^31^ ^]^ Additionally, the smaller size of the PNPs offers better potential for noninvasive applications and allows for easier and safer implementation into neural tissues compared to the PVDF membranes.

Other studies on bioactive nanostructures, including those by the groups of Stupp^[^ ^32^ ^]^ and Shoichet,^[^ ^33^ ^]^ have provided valuable insights into the role of bioactive materials and biochemical signals in NSC differentiation. The group of Stupp^[^ ^32^ ^]^ demonstrated that bioactive nanofibers presenting the neurite‐promoting laminin epitope IKVAV can induce selective neuronal differentiation from NSCs, underscoring the role of biochemical cues^[^ ^32^ ^]^ in this process. Meanwhile, the group of Shoichet^[^ ^33^ ^]^ highlighted the role of controlled release systems for delivering differentiation factors such as platelet‐derived growth factor (PDGF‐AA), emphasizing the relevance of polymer‐based nanosystems in guiding NSC differentiation. These cited studies reveal the impact of both biochemical and biophysical cues in guiding differentiation mechanisms.

Building on these advances, we propose the use of PNPs for piezoelectric stimulation to remotely trigger the neural differentiation of human neural stem cells (hNSCs). This approach combines the noninvasive benefits of US‐mediated stimulation with the high piezoelectric response of PNPs, offering a novel strategy for wireless, localized ES that has the potential to advance CNS regenerative therapies. Unlike earlier work that focused on PVDF membranes or biochemical signals, our study leverages P(VDF‐TrFE) nanostructures to provide targeted remotely‐activated stimulation, inducing neural differentiation through calcium signaling pathways that are independent of and synergic with traditional growth factor‐driven mechanisms.

## 2 Results and Discussions

### 2.1 PNP Characterization

**Figure 1:**
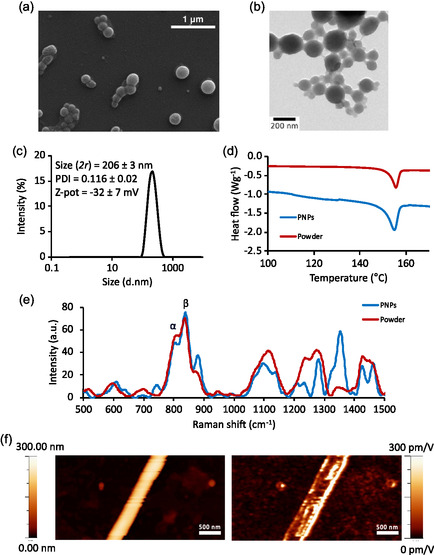
PNP characterization. Representative a) SEM and b) TEM images of PNPs; c) DLS analysis; d) DSC analysis; e) Raman spectroscopy; and f) PFM analysis: topography (left) and piezoelectric response (right) of the PNPs compared to a reference PVDF fiber.

The morphology of the PNPs was investigated by scanning electron microscopy (SEM) and bright‐field transmission electron microscopy (TEM). From the images shown in **Figure** **1**a,b, the spherical morphology of the nanoparticles can be observed. Dynamic light scattering (DLS) analysis (Figure 1c) allowed for the evaluation of the hydrodynamic diameter of the particles, which was measured to be 206 ± 3 nm, in perfect agreement with our previous work.^[^ ^34^ ^]^ The polydispersity index of 0.116 ± 0.02 and the *ζ*‐potential value of −32 ± 7 mV highlight the excellent stability of the nanoparticles, likely due to the residual presence of tween‐80, used as a stabilizer in the fabrication process. Indeed, washing and filtering the nanoparticles does not completely remove it.

Subsequent characterizations were carried out to investigate the crystallinity of the PNPs. Specifically, the percentage of crystallinity was assessed by differential scanning calorimetry (DSC) analysis, while Raman spectroscopy allowed the investigation of crystalline phase structures. The results obtained from these analyses were compared with those of the P(VDF‐TrFE) powder used to prepare the nanoparticles, to assess whether the particle preparation process favored the crystallization of the material and in which phase.

As reported in the literature, PVDF can crystallize into five crystalline structures: *α*, *β*, *γ*, *δ*, and *ε*. The most common are the first two, of which *α* is nonpolar and *β* is polar. For this reason, the latter is the most interesting for piezoelectric properties.^[^ ^29^, ^35^ ^]^ However, PVDF tends to crystallize in the *α* phase, and poling is necessary to achieve recrystallization in the *β* phase. Conversely, in P(VDF‐TrFE), the presence of the trifluoroethylene monomer (CHF = CF_2_) promotes the formation of a phase similar to the *β* phase, which is polar and thus suggestive of piezoelectric properties.^[^ ^29^ ^]^

Figure 1d shows the DSC graph comparing the melting peaks (≈155 °C) of P(VDF‐TrFE) powder and PNPs. The presented thermograms only depict the temperature range around the nanoparticle melting peak, to illustrate the crystallinity of the nanoparticles. From this analysis, it can be concluded that the fabrication process of the PNPs induces an increase in material crystallinity, with a peak enthalpy value shifting from −11.28 to −24.16 J g^−1^. For the calculation of the percentage of crystallinity, these values were compared with the melting enthalpy associated with 100% crystallinity reported in the literature for P(VDF‐TrFE,) equal to 38 J g^−1^,^[^ ^36^ ^]^ resulting in 29.69% and 63.57% of crystallinity for the powder and the nanoparticles, respectively.

Raman spectroscopy allowed for the evaluation of the different crystalline structures, aiming to highlight any changes in the distribution of the various phases between powder and nanoparticles, and to confirm the presence of the *β* phase in the PNPs. As known from the literature,^[^ ^34^ ^]^ the *α* phase is characterized by a peak at 803 cm^−1^ and the *β* phase by a peak at 848 cm^−1^. From the graph in Figure 1e, these two peaks can be observed, along with an additional peak at 880 cm^−1^, which arises from the contribution of *α*, *β,* and *γ* phases.^[^ ^37^ ^]^ Compared to the powder, the PNPs display a slight increase in the peak at 880 cm^−1^, which includes contributions from the piezoelectric *β* phase as well as the from the *α* and *γ* phases. These results are in line with the overall increase in crystallinity observed through DSC analysis and demonstrate that the increase in crystallinity in PNPs pertains to the polar phases of the material.

Lastly, the piezoelectric properties of nanoparticles were tested. To this end, piezoresponse force microscopy (PFM) analysis was performed on PNPs. Following the application of an EF between the probe (used in noncontact mode, in constant‐excitation frequency modulation [CE–FM]) and the substrate, the nanoparticles responded with a piezo‐displacement, thus exhibiting the expected piezoelectric properties. Piezoelectric coefficient *d*_33_ resulted 17.3 ± 9.3 pm V^−1^ and was compared to the *d*_33_ of a reference PVDF fiber, 14.4 ± 4.5 pm V^−1^. Further details and all the measurements are reported in Table S1, Supporting Information. Figure 1f shows the topography (left) and the piezoelectric features (right) of the PNPs. The piezoelectricity characterization is consistent with the detected crystalline phase; indeed, the presence of a rather high percentage of crystallinity and the polar *β* phase are suggestive of potential piezoelectricity, confirmed by PFM. It is also noteworthy that the piezoelectric coefficients measured on nanoparticles are even slightly higher than those measured on the control PVDF fiber, further confirming that the piezoelectric is enhanced in the copolymer, as already reported in the literature.^[^ ^29^ ^]^

### 2.2 PNP In Vitro Assessment

**Figure 2:**
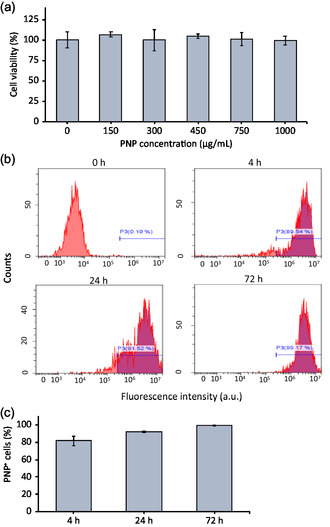
PNPs/cells interactions. a) WST‐1 assay to assess PNP cytocompatibility; b) representative flow cytometry data concerning internalization; and c) quantitative assessment of the internalization.

The PNP cytocompatibility was assessed using the WST‐1 assay at different concentrations: 0, 150, 300, 450, 750, and 1000 μg mL^−1^. As evident from the graph in **Figure** **2**a, after 72 h of exposure to PNPs at all the tested concentrations, hNSCs continue to exhibit a level of viability comparable to the control. The following experiments have been performed by using a safe concentration of 450 μg mL^−1^.

Cell internalization assessment was performed using fluorescently‐labeled PNPs at different incubation times (0 [as reference], 4, 24, and 72 h). Figure 2b shows representative flow cytometry experiments, indicating a high extent of internalization already after 4 h of incubation. Indeed, the overall quantitative evaluation (Figure 2c) highlighted already 82% PNP^+^ cells upon a 4 h of incubation, a value that almost reaches 100% at the end of the observation period (72 h).

Further analyses were performed to investigate the interaction between PNPs and cells. A first experiment evaluated the co‐localization between PNPs and lysosomes. Indeed, it is important to verify that nanoparticles are not uptaken in cell organelles aimed at their elimination, to ensure that they can perform their function. As shown in Figure S1a, Supporting Information, the co‐localization of PNPs increases overtime. It is indeed expected that as particles are internalized, some will be uptaken by lysosomes, and this is evident in the merged images, where the yellow signal (overlap between lysosomes in red and PNPs in green) is virtually absent at 4 h, slightly present at 24 h, and more pronounced at 72 h. The quantitative analysis provided in Figure S1b, Supporting Information, shows that despite a consistent increase of lysosomal internalization occurs, at 72 h just 24% of the PNPs are internalized in these organelles, thus being most of them free in the cytoplasm or associated to other cellular structures.

The same analysis was carried out to evaluate the co‐localization with actin, to have a hint of PNP/cell association. Indeed, PNP internalization is not essential for our goals, and high cell/nanotransducers association is sufficient for eliciting electric stimulation in cells.^[^ ^30^, ^38^ ^]^ Figure S2a, Supporting Information, shows that after 4 h of incubation, the nanoparticle signal (green) is still weak and scattered, not closely associated with the cytoskeleton signal (red actin). At 24 h, there is a clear co‐localization of the two signals, which further increases at 72 h. Quantitative evaluation (Figure S2b, Supporting Information) shows the cell area occupied by nanoparticles at different time points: it can be observed that the trend increases overtime as the particles are internalized, which is consistent with the results of the flow cytometry analysis (Figure 2c). At 72 h, almost 40% of the cell area is occupied by PNPs: this analysis provides some important information: 1) high co‐localization implies a strong cell–nanoparticle interaction (whether external or internal to the cell); 2) higher co‐localization with respect to lysosomes imply that nanotransducers are not processed by the digestive organelles of the cells and thus can efficiently accomplish their transduction task upon US stimulation.

### 2.3 Acute Piezoelectric Stimulation

The effect of PNP‐mediated stimulation was investigated through calcium imaging. After calcium labeling using the Fluo‐4 AM dye, cells were stimulated by US both in the absence and in the presence of nanoparticles. This analysis enables assessing whether an increased calcium‐mediated activation occurs during stimulation. This is important because calcium plays a critical role in the regulation of many cell processes, including those related to neuronal differentiation and function.^[^ ^39^ ^]^ During neuronal differentiation, fluctuations in intracellular calcium levels occur, which can regulate gene expression, synapse formation, and neuronal morphology.^[^ ^39^ ^]^ As NSCs differentiate, the expression of genes responsible for maintaining the pluripotent phenotype is repressed, while genes conferring typical neuronal properties are more widely expressed.^[^ ^39^ ^]^ Several factors promote neuronal differentiation, such as NeuroD: like many others, this factor can only act after activating certain calcium‐dependent pathways; indeed, it is phosphorylated by the enzyme CaMK, the activity of which depends on intracellular calcium.^[^ ^39^ ^]^ Calcium is also crucial for neuronal development and plasticity, which is regulated by the number and frequency of calcium transients; in particular, calcium spikes, which involve a rapid increase in calcium concentration generated by calcium‐dependent action potentials and calcium‐induced calcium release, propagate rapidly throughout the cell and are primarily responsible for regulating neurotransmitter expression. Calcium waves, in contrast, are variable phenomena in terms of duration and shape, and can form in both the cell body and the growth cone, but tend not to propagate and remain confined to the cell compartment where they originate.^[^ ^40^ ^]^ Their function is more closely related to the axonal development and the growth and branching of dendritic trees, which influence the number of synaptic connections developed by neurons.^[^ ^40^ ^]^

**Figure 3:**
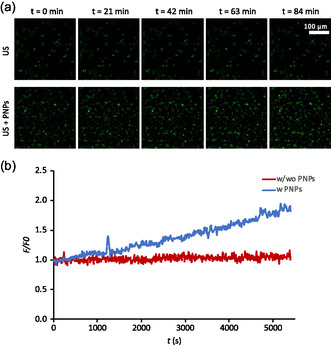
Calcium imaging during US stimulation, in presence or absence of PNPs. a) Representative frames from the time lapse; b) quantitative analysis.

**Figure** **3**a reports representative images of the acquired time lapse, acquired either in the presence or absence of PNPs, during US stimulation. In the first case, no significant cell activation is visible; conversely, when cells undergo the US + PNP stimulation, a significant response in terms of calcium flows is clearly detected. Figure 3b reports the quantitative analysis of this test, showing the evolution of the calcium signal overtime, normalized to the value at *t* = 0 *(F/F*_0_). Once again, we have the confirmation that, in the absence of PNPs, the calcium signal remains constant throughout the stimulation period. Conversely, in the presence of PNPs, an increasing trend can be observed, suggestive of an increased cellular activity.

The observation of calcium differential signals during stimulation shows that cells are activated in the presence of PNPs upon US stimulation, and it gives an important hint about an effective mechano‐electric transduction.

### 2.4 hNSC Differentiation upon Chronic Piezoelectric Stimulation

Neuronal differentiation after chronic piezoelectric stimulation (1 h day^−1^ for 3 days) was assessed by immunocytochemical evaluation of two neuronal markers, NeuN and βIII‐tubulin. High expression of NeuN is associated with neuronal maturity, whereas βIII‐tubulin is predominantly expressed in the neuronal cytoskeleton of immature neurons and is an indicator of neuronal growth and neurite outgrowth.^[^ ^41^ ^]^

Over the last decade, the NeuN protein has emerged as a widely used marker for studying the differentiation of stem cells into neurons.^[^ ^42^ ^]^ While various marker proteins such as βIII‐tubulin, MAP‐2, doublecortin, synaptophysin, and others have been used to identify nervous system cells, NeuN offers distinct advantages. Unlike some markers that are also found in nonneuronal cells, NeuN is expressed exclusively in the nervous tissue.^[^ ^42^ ^]^ In addition, NeuN is absent from immature neural progenitors and is primarily associated with the nucleus, making it ideal for detecting mature neurons.^[^ ^42^ ^]^ Moreover, from a technical point of view, this nuclear localization, conversely to cytoplasmic markers, facilitates precise staining and image processing for automated quantitative analysis. NeuN thus represents a reliable and specific post‐mitotic marker for neuronal differentiation studies.^[^ ^42^ ^]^

**Figure 4:**
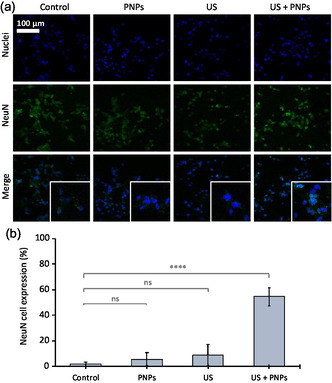
NeuN expression investigation. a) Representative confocal images; b) quantitative analyses (****p < 0.001).

Representative images for each experimental class are shown in **Figure** **4**a, while the quantitative evaluation is provided in Figure 4b. The percentage expression in the control is very low (1.8% ± 1.6%), as expected in non‐differentiated cells. Furthermore, although Figure 4b might suggest an increasing trend for the PNPs and US treatments, possibly attributable to a mechanical stimulation of cells either mediated by US or following PNP internalization,^[^ ^43^, ^44^ ^]^ this increase is not significant (5.6% ± 5.2% and 8.7% ± 8.5%, respectively).^[^ ^43^, ^44^ ^]^ NeuN expression in cultures stimulated with US in the presence of nanoparticles is conversely significantly higher (54.6% ± 7.3%), a result that suggests an extensive effect of the piezo‐stimulation.

**Figure 5:**
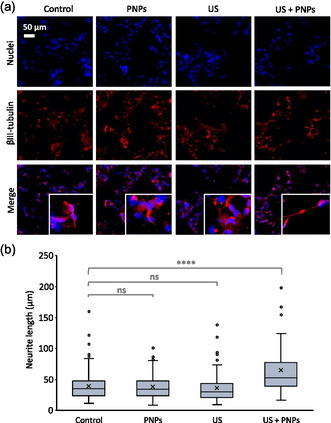
Evaluation of neurite development following piezo‐stimulation in proliferative conditions. a) Representative confocal images of βIII‐tubulin immunostaining; b) neurite length distribution (****p < 0.001).

Immunofluorescence analyses were carried out also for the detection of βIII‐tubulin. This protein is peculiar of the neuronal cytoskeleton and is characteristic of an early stage of neuronal differentiation. Being an early marker, βIII‐tubulin is also expressed in NSCs and no major difference in the expression of this marker is expected after neuronal differentiation (**Figure** **5**a). However, as βIII‐tubulin is associated with microtubules, it is a neuritic marker and can therefore be exploited to compare the length of neurites in the different experimental classes. Indeed, increased axonal extension promotes cell communication and is an indicator of neuronal development. Analyses of the effect of chronic piezoelectric stimulation on axonal growth were performed on hNSCs in proliferation medium and in differentiation medium to assess a possible effect of a combined physical–chemical stimulus.

Figure 5a shows the confocal images of hNSCs in proliferation medium in the different experimental classes after βIII‐tubulin immunostaining. As expected, the signal intensity of the protein does not vary significantly among the different experimental classes. However, it is evident an increase in neurite length in the cultures undergoing piezoelectric stimulation. These qualitative assessments are confirmed by the quantitative analyses shown in the box plots of Figure 5b. In particular, it appears that neurite length is comparable for the first three experimental classes (control, PNPs, and US), while the distribution shifts to higher values for the US + PNPs experimental class (53.17 ± 7.4 μm).

**Figure 6:**
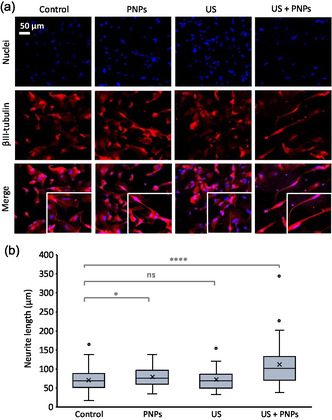
Evaluation of neurite development following piezo‐stimulation in differentiative conditions. a) Representative confocal images of βIII‐tubulin immunostaining; b) neurite length distribution (****p < 0.001, *p < 0.05).

Similar results are observed for hNSCs in differentiation medium (**Figure** **6**a). The box plots in Figure 6b show a significant difference between the neurite length of piezoelectrically stimulated hNSCs (US + NPs) and the other three experimental classes. Again, the neurite length significantly increases after stimulation from 68.00 ± 5.46, 76.00 ± 6.14, and 69.00 ± 6.04 μm for the first three experimental classes (control, PNPs, and US, respectively) to 102.00 ± 13.37 μm for the US + PNPs class. Although not very pronounced, a significant difference can be observed between the axonal length distribution of the control and of the PNP treatment. Further studies are needed to verify whether there is indeed an effect in this condition, yet this result would be consistent with what has been reported in some studies in the literature, where the mere presence of the piezoelectric material can induce the growth of neuritic extensions,^[^ ^43^ ^]^ enhanced in presence of the differentiative biochemical factors. Overall, the presence of the differentiative medium induces chemical differentiation also in otherwise unstimulated control cells, as expected, and this effect is additive to the piezo‐stimulation in the US + PNPs experimental class.

### 2.5 Gene Expression Regulation upon Chronic Piezoelectric Stimulation

To investigate the growth factor‐independent mechanisms of piezoelectric stimulation‐induced neural differentiation, we performed real‐time reverse‐transcription polymerase chain reaction (RT‐PCR) gene expression analysis on *c‐Jun*, *c‐Fos*, *neurogenic differentiation 1* (*NeuroD1*), and *laminin subunit beta‐1* (*Lamb1*) after 3 days of chronic stimulation. Results were then compared to the control classes. *c‐Jun* and *c‐Fos* genes encode transcription factors that are part of the activator protein‐1 complex, which regulates gene expression in neural proliferation and differentiation,^[^ ^45^ ^]^ particularly in response to neurotrophic factors like NGF.^[^ ^46^ ^]^ *NeuroD1* gene encodes for a master transcription factor that plays a pivotal role in neural differentiation, guiding the expression of multiple genes involved in neuronal identity, growth, and maturation.^[^ ^47^, ^48^ ^]^ *Lamb1* is a late gene that encodes a key component of the laminin‐1 protein, which plays a crucial role in the extracellular matrix, especially in the nervous system.^[^ ^49^ ^]^ *Lamb1* expression is essential for neural development processes like neuronal migration, neurite outgrowth, and synapse formation. *Lamb1* is gradually upregulated as the neuronal differentiation process proceeds.^[^ ^49^ ^]^

**Figure 7:**
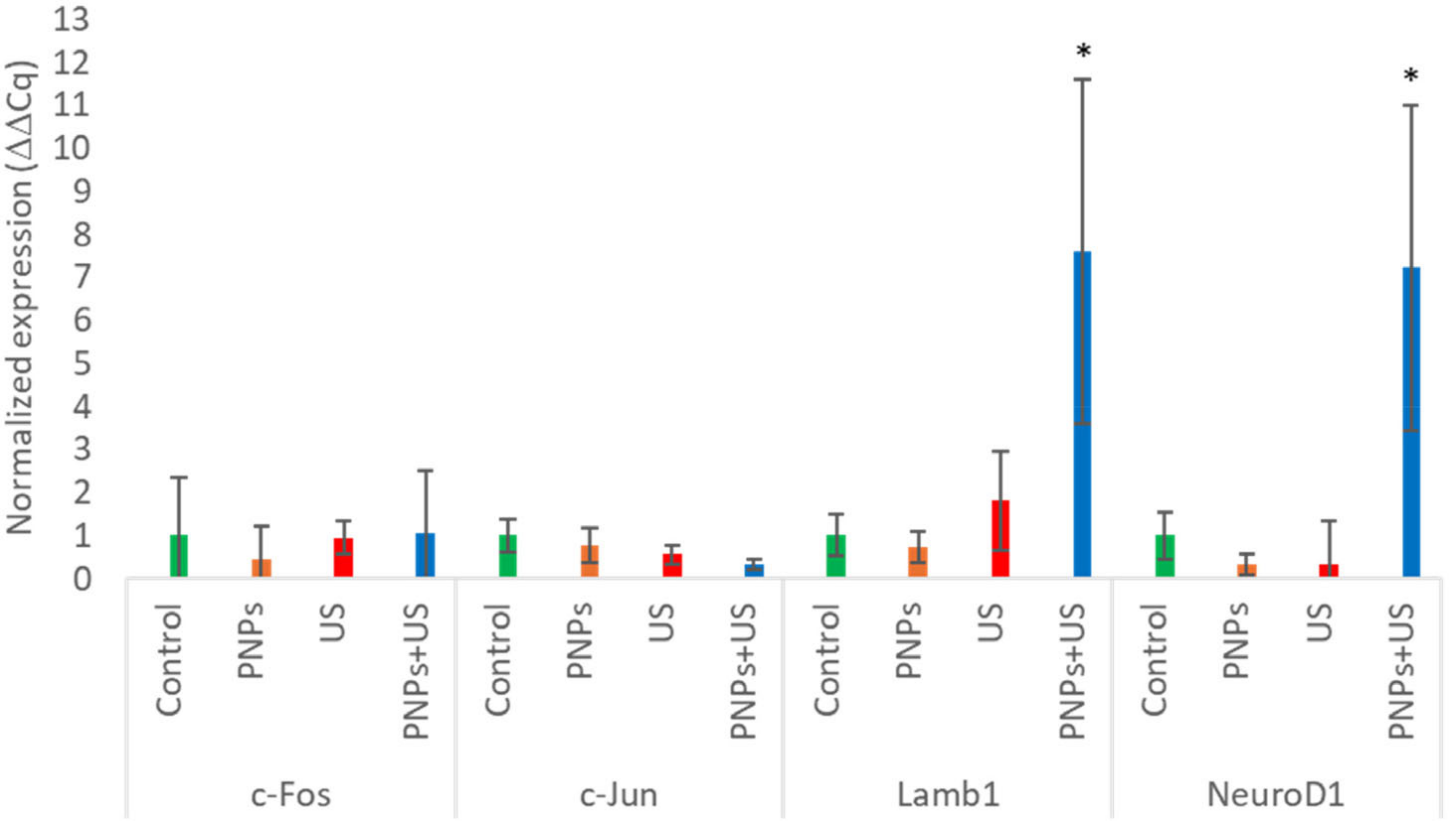
Relative mRNA quantification of *c‐Fos*, *c‐Jun*, *Lamb1*, and *NeuroD1* genes in control, PNPs, US, and PNPs + US conditions, normalized to the control; *p < 0.05.

Overall, RT‐PCR results (**Figure** **7**) demonstrated a significant upregulation of *NeuroD1* (7.22 ± 3.79‐fold) and *Lamb1* (7.61 ± 3.99‐fold) in the hNSCs chronically stimulated with US + PNPs, while no significant effects were observed for *c‐Fos* (1.04 ± 1.46‐fold) and *c‐Jun* (0.32 ± 0.11‐fold) with respect to the control. No remarkable upregulation with respect to the control cultures (in all the following cases *p* > 0.05) was detected in the case of PNP treatment without US application (0.76 ± 0.40‐fold for *c‐Jun*, 0.46 ± 0.75‐fold for *c‐Fos*, 0.34 ± 0.25‐fold for *NeuroD1*, 0.72 ± 0.36‐fold for *Lamb1*) and in the case of US stimulation in the absence of PNPs (0.55 ± 0.22‐fold for *c‐Jun*, 0.95 ± 0.40‐fold for *c‐Fos*, 0.33 ± 1.02‐fold for *NeuroD1*, 1.80 ± 1.14‐fold for *Lamb1*), indicating the requirement of both US and PNPs for the successful induction of neuronal differentiation. Overall, the RT‐PCR results suggest that piezoelectric stimulation‐induced differentiation is mediated by the upregulation of both early (*NeuroD1*) and late (*Lamb1*) pro‐neural genes, without the involvement of *c‐Fos*/*c‐Jun* regulation. This may be attributed to the ability of Ca^2+^ elevations to upregulate *NeuroD1* through pathways independent of *c‐Fos*/*c‐Jun*, such as the Wnt/β‐catenin pathway, which is known to inhibit GSK3 and ultimately induce the upregulation of *NeuroD1*.^[^ ^50^ ^]^ In this context, alternative stimulation methods, like those involving EMFs, can promote neural differentiation and neuritogenesis by upregulating *NeuroD1* via Ca^2+^ influx.^[^ ^50^, ^51^, ^52^ ^]^

## 3 Conclusion

This work aimed to use PNPs to promote neuronal differentiation of hNSCs through chronic piezoelectric stimulation. Indeed, electrical cues are known to be crucial for hNSC differentiation.^[^ ^16^ ^]^ The remote activation of the proposed nanotransducers allows the delivery of the electrical stimulus without the need for a direct approach, thus avoiding electrode‐associated issues. Furthermore, the ability to use synthetic polymers as piezoelectric nanotransducers facilitates the potential clinical translation of the platform and paves the way for the use of other synthetic piezoelectric polymers with greater biological applicability (e.g., biodegradable polymers). The differentiation of hNSCs resulted remarkably improved upon piezostimulation, both in proliferative and differentiative biochemical cues. Future research will aim to evaluate the capacity of our stimulation approach to enhance the electrophysiological maturation of the obtained post‐mitotic neurons, ensuring their membranes become fully electroresponsive. This aspect will be particularly significant for promoting NSC differentiation in vivo, where functional integration is critical. In contrast, when differentiation occurs in vitro with the aim of subsequent grafting, it is important for complete maturation and the formation of synaptic connectivity to be achieved post‐implantation.^[^ ^53^ ^]^ Therefore, while our current study lays the groundwork for understanding these processes, the elucidation of electrophysiological mechanisms will be reserved for future investigations.

## 4 Experimental Section

4.1

4.1.1

##### PNP Synthesis

PNPs were prepared according to the protocol previously reported by our group.^[^ ^34^ ^]^ Specifically, 20 mg of P(VDF‐TrFE) (FC45, Piezotech) were dissolved in acetone (Sigma Aldrich) at a concentration of 5 μg mL^−1^. This solution was slowly dripped using a 0.7 mm needle into a tween‐80 (Sigma‐Aldrich) water solution (0.2% w/v, 9 mL) under moderate stirring (350 rpm); the solution was left under stirring for 2 h at room temperature. It was then collected, avoiding the recovery of aggregates at the bottom, and sonicated for 10 min in an ice bath (amplitude 70%) using an ultrasonic tip (Fisherbrand Q125 Sonicator). To remove acetone and, partially, tween‐80, five washes were performed in MilliQ water with Amicon centrifuge filters (Ultra‐4 Centrifugal Filter Unit, MWCO 100 kDa, Sigma‐Aldrich) at 4000 g for 10 min at 25 °C. Finally, the particles were resuspended in sterile MilliQ water and filtered through a 1.2 μm filter to remove aggregates.

For fluorescent PNP synthesis, 10 μL of VybrantTM DiO (Invitrogen) were added to the initial acetone solution (4 mL).

##### PNP Characterization

SEM analysis was performed using a Helios NanoLab 600i FIB/SEM, FEI. A drop (5 μL) of the sample at a concentration of 50 μg mL^−1^ was poured onto a silicon wafer and allowed to dry. The sample was then gold‐sputtered using a Quorum Tech Q150RES gold sputter coater at 10 mA for 30 s and imaging was performed.

For TEM analysis, a drop of the dispersion (concentration of 50 μg mL^−1^) was deposited on a Cu grid (150 mesh) coated with an ultrathin amorphous carbon film and allowed to dry at room temperature for 2 h. TEM analyses were carried out with a JEM‐1400Plus, operating at 120 kV.

The hydrodynamic size and ζ‐potential were assessed using a Zeta‐sizer NanoZS90 (Malvern Instruments Ltd). Measurements were performed on dispersions at a concentration of 100 μg mL^−1^ in ultrapure MilliQ water. CONTIN analysis was exploited to extract the intensity distribution and cumulant analysis to determine the hydrodynamic diameter and polydispersity index.

DSC curves were generated utilizing a DSC‐1 STARe System (Mettler Toledo). The analysis was conducted on a few milligrams (1.6 mg) of freeze‐dried samples, scanning from 20 to 200 °C at a heating rate of 10 °C min^−1^. The melting peak area value, which corresponds to the fusion enthalpy of the nanoparticles, was normalized to the melting enthalpy associated with 100% crystallinity of the material to calculate the crystallinity percentage of the nanoparticles.

Raman spectroscopy was performed on both polymer powder and on PNPs to assess peak variations and correlate them with a change in the crystalline phases. Raman analysis was performed using the LabRAM HR Evolution microscope by Horiba, equipped with a 532 nm laser. Specifically, a 100× objective was used, and the laser power was set to 3.2% of its maximum output. Data were acquired between 500 and 1500 cm^−1^ of Raman shift, with an acquisition time of 2 s and 10 repetitions.

The piezoresponse of individual nanoparticles was evaluated using a PFM technique, based on atomic force microscopy (AFM), named CE–FM–PFM. In this method, intermittent contact mode AFM was used to reduce the average AFM probe force of conventional PFM performed in AFM contact mode. This method was designed to allow operation on soft materials and nanostructures loosely attached to the substrate. Piezoresponse detection is achieved by polarizing the sample with an oscillating electric potential (80 Hz frequency) through the conductive probe, followed by demodulation of the resulting force. The technique was implemented on a NanoScope IIIa AFM with MultiMode head, featuring ADC5 extension (Veeco Instruments Inc.), in combination with a phase‐locked‐loop controller (PLLProII, RHK Technology), and a dual‐phase lock‐in amplifier (SRS830DSP, Stanford Research Systems) for piezoresponse demodulation. Non‐contact mode conductive AFM cantilevers were utilized (Nanosensors, model PPP‐NCLPt, platinum–iridium‐coated silicon tips, spring constant ≈40 N m^−1^, resonant frequency ≈156 kHz, quality factor ≈500 in air, tip radius ≈30 nm). The adopted free oscillation amplitude of the cantilever was ≈20 nm. Image processing was performed using WSxM freeware, version 4 beta 9.3 (wsxm.eu).^[^ ^54^ ^]^

##### In Vitro Testing

Human neural hindbrain stem cells (Y40060), purchased from Takara Bio Inc., were used for the experiments. Cells were seeded at a density of 3.5 × 10^4^ cm^−2^ in laminin‐coated T25 flasks (2 mL of 10 μg mL^−1^ laminin in phosphate‐buffered saline (PBS) for 3 h at 37 °C). Human NSCs were cultured in RHB‐A medium (Takara Bio Inc.) supplemented with 100 μg mL^−1^ streptomycin (Gibco), 20 ng mL^−1^ recombinant human epidermal growth factor (EGF, Peprotech), and 20 ng mL^−1^ recombinant human fibroblast growth factor (FGF‐2/bFGF, Peprotech) at 37° in 5% CO_2_ fully humidified atmosphere. The culture medium was changed on alternate days, and cells were split 1:2 approximately every 7 days, after reaching 80%–90% confluence in the flask. Cultures up to passage P6 were used for experiments.

For cell experiments in differentiation medium, a poly‐L‐ornithine coating (Sigma‐Aldrich, 0.01% w/v) was performed for 20 min at room temperature before laminin coating. The employed differentiation medium consists of RHB‐basal medium (Takara Bio Inc.), supplemented with 100 μg mL^−1^ streptomycin (Gibco), 0.5% NDiff N2‐AF (Takara Bio Inc.), 0.5% NDiff N27 (Takara Bio Inc.), and 10 ng mL^−1^ recombinant human bFGF (Peprotech).

Cell viability was assessed using the WST‐1 assay ((2‐(4‐iodophenyl)‐3‐(4‐nitrophenyl)‐5‐(2,4‐disulfophenyl)‐2H‐tetrazolium monosodium salt, BioVision) to determine the metabolic activity after incubation with nanoparticles at different concentrations, and identify the highest nontoxic concentration for cell stimulation experiments. The experiment included six different concentrations of PNPs (0, 150, 300, 450, 750, and 1000 μg mL^−1^). For this purpose, a 96‐well plate was used, and six wells were seeded for each concentration (100 μL of PNP‐doped proliferation medium per laminin‐coated well). The WST‐1 analysis was carried out after 72 h of incubation; the culture medium was removed and replaced with WST‐1 diluted 1:11 in DMEM (100 μL well^−1^) supplemented with 10% fetal bovine serum. The multiwell plate was then incubated for 40 min, and afterward, absorbance was read at 450 nm using a microplate reader (Victor3, PerkinElmer). The metabolism of the different experimental conditions was then expressed by normalizing the absorbance values to those of the control cultures.

Cell internalization analyses were performed with fluorescent PNPs (450 μg mL^−1^) at three different time points: 4, 24, and 72 h. The analyses were performed using a flow cytometer (CytoFLEX, Beckman Coulter) to assess the percentage of cells positive for fluorescence (*λ*_ex_ 488 ± 40 nm, *λ*_em_ 525 ± 40 nm). The autofluorescence threshold was determined from control data (0 h of PNP exposure). Data were processed using the CytoFLEX software.

Cell internalization was also confirmed with confocal microscopy imaging (C2s confocal microscope, Nikon) on cultures treated as stated earlier.

Co‐localization with lysosomes was performed again in the same conditions with confocal microscopy, by treating cultures with 1:1000 v v^−1^ LysoTracker Deep Red dye (Invitrogen) for 50 min. Cell nuclei were counterstained with 0.5 μL of Hoechst 33 342, following a 10 min incubation.

For f‐actin detection, cultures were fixed (4% w/v paraformaldehyde at 4 °C for 20 min) and treated for 30 min with TRITC–phalloidin (1:200) and Hoechst 33 342 (1:1000). Zoomed‐in inlets were provided in the confocal merged figures to emphasize the differences among the experimental classes.

##### Piezoelectric Stimulation

Calcium imaging was performed to monitor the real‐time neuronal activity of cells undergoing acute US stimulation and to compare the effects of mechanical stimulation alone with those mediated by PNPs. Human NSCs were seeded on laminin‐coated μ‐Plate 24‐well plates (Ibidi) at 50% of confluence. Four experimental classes were considered: control, PNPs, US, and US + PNPs; PNP concentration was set at 450 μg mL^−1^. Twenty‐four hours after treatment, US stimulations were performed using a KTAC‐4000 device (Sonidel) equipped with a planar US transducer (20 mm diameter) operating at a power of 1 W cm^−2^, a frequency of 1 MHz, a burst rate of 0.5 Hz and a duty cycle of 10%. These specific US parameters were chosen to avoid significant temperature increases in the cell medium, even during prolonged stimulation, as previously demonstrated.^[^ ^55^ ^]^ Cells were stimulated for 1 h; calcium imaging was started 15 min before the stimulation and was ended 15 min after the end of stimulation. At this aim, cells were incubated with Fluo‐4 AM (1 μM, Invitrogen) at 37 °C for 40 min prior to US stimulation. The samples were then rinsed with PBS and incubated with phenol red‐free DMEM supplemented with HEPES (25 mM, Thermo Fisher) for time‐lapse fluorescence imaging using an Eclipse Ti‐E epifluorescence microscope (Nikon). Images were acquired at a rate of one every 10 s. Fluorescence intensities at different time points were calculated as the mean value of pixels measured in the intracellular region of interest, with the extracellular background being subtracted. The graphs show the *F/F*_0_ (being *F* the average fluorescence intensity and *F*_0_ its value at *t* = 0) curves corresponding to different experimental conditions (US and US + PNPs) during the time‐lapse experiment. Representative images at *t* = 0, 21, 42, 63, and 84 min were selected for both experimental classes.

Chronic stimulation experiments were carried out in both proliferation and differentiation media, to assess whether the combination of biochemical and biophysical factors favors differentiation to a greater extent. Cells were seeded in a μ‐Plate 24‐well plate (Ibidi, at 50% confluence); for stimulation in proliferation conditions, cells were seeded in proliferation medium after laminin coating, while for stimulation differentiation conditions, cells were seeded in differentiation medium after poly‐L‐ornithine coating (250 μL well^−1^ for 20 min at room temperature, 0.01% w/v) and laminin coating (250 μL well^−1^ of 10 μg mL^−1^ laminin for 3 h at 37 °C). Four experimental classes were considered: control, PNPs, US, and US + PNPs; PNP concentration was set at 450 μg mL^−1^. In US‐treated groups, trains of US were delivered at an intensity of 1 W cm^−2^ and a frequency of 1 MHz. Single stimuli were activated every 2 s and lasted 200 ms each. Stimulation was repeated for 3 days, 1 h day^−1^. At the end of the experiments, cells were fixed in PFA as previously described.

The effect of chronic stimulations on hNSC differentiation was assessed by monitoring the expression of two neuronal markers (NeuN and βIII‐tubulin) through immunofluorescence. After fixation, membrane permeabilization (with Triton X‐100 in 0.1% PBS for 1 h at room temperature), and treatment with a blocking solution (10% goat serum in PBS for 1 h), the cultures were incubated with primary antibodies: mouse IgG anti‐NeuN (diluted 1:300 in 10% goat serum for 2 h at 37 °C; Merck) or rabbit IgG anti‐βIII‐tubulin (diluted 1:300 in 10% goat serum for 2 h at 37 °C; Merck). Subsequently, the cells were washed twice with 10% goat serum in PBS and incubated with a staining solution consisting of 10% goat serum in PBS containing Hoechst 33 342 (1:300, Invitrogen) and the secondary antibody (anti‐mouse or anti‐rabbit, 1:250, Invitrogen). Images were acquired with the confocal microscope and analyzed with Image J. Zoomed‐in inlets have been provided in the confocal merged figures to emphasize the differences between the experimental classes.

##### Gene Expression Analysis in Response to Piezoelectric Stimulation

Chronic piezoelectric stimulation experiments were conducted under proliferative conditions to investigate growth‐factor‐independent pathways involved in neural differentiation induction. Following the chronic piezoelectric stimulation of the cells, the transcriptional activity of *c‐Jun*, *c‐Fos*, *NeuroD1*, and *Lamb1* was assessed in control, PNPs, US, and US + PNPs experimental classes through quantitative real‐time qRT‐PCR. mRNA was isolated and purified using the RNeasy Kits (Qiagen) according to the manufacturer's guidelines. To quantify mRNA, 1 μL from each experimental class was analyzed using a NanoDrop (Thermo Scientific) for spectrophotometric measurements. Reverse transcription was performed with 100 ng of RNA from each sample using the iScript Advanced cDNA Synthesis Kit for qRT‐PCR (Bio‐Rad), converting the mRNA into complementary DNA (cDNA). The thermal cycling conditions for reverse transcription were 46 °C for 20 min, followed by 95 °C for 1 min. Amplification of the cDNA was carried out in a CFX Connect Real‐Time PCR Detection System (Bio‐Rad) using the SsoAdvanced Universal SYBR Green Supermix (Bio‐Rad). The amplification program included an initial temperature step at 95 °C for 30 s for polymerase activation and DNA denaturation, followed by 50 cycles at 98 °C for 10 s and 60 °C for 20 s. A final step involved a melting curve analysis, where the temperature increased from 65 to 95 °C at a rate of 0.5 °C s^−1^. For normalization, *β actin* was used as reference genes. The cycle threshold (*Ct*) values of the control (cultures without nanoparticle incubation and US stimulation) served as the baseline for calculating ΔΔ*Ct* in the experimental conditions. Gene modulation >fivefold or <0.2‐fold compared to the control, along with *p* < 0.05, was used to determine upregulation and downregulation, respectively. The sequences of the primers used (both forward and reverse) for the targeted genes are detailed in Table S2, Supporting Information.

##### Statistical Analysis

The normality of distributions was initially assessed using the Shapiro test. For normal distributions (i.e., cell internalization and NeuN expression), one‐way ANOVA was carried out to evaluate significant differences among experimental classes. Bonferroni's test was performed as post hoc test for pairwise comparisons between experimental classes (significance was attributed for ****p < 0.001 and *p < 0.05). For non‐normal distributions (i.e., neurite length), the median value and the 95% confidence interval (CI) were reported, with the latter calculated according to Equation (1) (95% CI; interquartile range; *N*: number of considered neurites *per* experimental class). Kruskal–Wallis test was performed for statistical analysis for non‐normal distributions. Dunn's test was then implemented for pairwise comparisons among the experimental classes.

(1) $$\text{CI}=1.58\times \frac{\text{IQR}}{\sqrt[{2}]{N}}$$

## Conflict of Interest

The authors declare no conflict of interest.

## Author Contributions

**Arianna Bargero**: Data Curation (lead); Formal Analysis (equal); Investigation (lead); Validation (equal); and Writing—Original Draft (lead). **Matteo Battaglini**: Data Curation (equal); Formal Analysis (equal); Investigation (equal); and Methodology (equal). **Tommaso Curiale**: Data Curation (supporting); Investigation (supporting); and Methodology (supporting). **Alessio Carmignani**: Data Curation (equal); Formal Analysis (equal); Investigation (equal); and Methodology (equal). **Margherita Montorsi**: Data Curation (supporting); Formal Analysis (supporting); Investigation (supporting); and Methodology (supporting). **Massimiliano Labardi**: Data curation (equal); Formal analysis (equal); Investigation (lead); Methodology (lead); Methodology (lead); and Validation (equal). **Carlotta Pucci**: Data Curation (supporting); Formal Analysis (supporting); Investigation (supporting); and Methodology (supporting). **Attilio Marino**: Conceptualization (equal); Data Curation (equal); Formal Analysis (equal); Investigation (lead); Methodology (lead); Supervision (equal); Validation (lead); and Writing—Original Draft (lead). **Gianni Ciofani**: Conceptualization (equal); Project Administration (lead); Resources (lead); Supervision (equal); and Writing—Review and Editing (lead).

## Supporting information

Supplementary Material

## Data Availability

The data that support the findings of this study are available from the corresponding author upon reasonable request.
