# Supplementary material for: Ultrasound‐Responsive Polymeric Piezoelectric Nanoparticles for Remote Activation and Neuronal Differentiation of Human Neural Stem Cells
Source: Small Sci. 2024 Nov 27;5(2):2400354. doi: 10.1002/smsc.202400354 (PMC11934905; doi:10.1002/smsc.202400354)
Supplement: Supplementary file 1 — Supplementary Material [file SMSC-5-2400354-s001.pdf]

Supporting Information

Ultrasound-Responsive Polymeric Piezoelectric Nanoparticles for Remote Activation and Neuronal Differentiation of Human Neural Stem Cells

Arianna Bargero, Matteo Battaglini, Tommaso Curiale, Alessio Carmignani, Margherita Montorsi, Massimiliano Labardi, Carlotta Pucci, Attilio Marino, Gianni Ciofani**

Attilio Marino and Gianni Ciofani equally contributed to this work as last-co-authors.

A. Bargero, M. Battaglini, T.Curiale, A. Carmignani, M. Montorsi, C. Pucci, A. Marino*, G. Ciofani*
Istituto Italiano di Tecnologia, Smart Bio-Interfaces, Viale Rinaldo Piaggio 34, 56025 Pontedera, Italy
gianni.ciofani@iit.it
attilio.marino@iit.it

A. Bargero, T. Curiale
Politecnico di Torino, Department of Mechanical and Aerospace Engineering, Corso Duca degli Abruzzi 24, 10129 Torino, Italy

M.Montorsi
Scuola Superiore Sant'Anna, The Biorobotics Institute, Viale Rinaldo Piaggio 34, 56025 Pontedera, Italy

M. Labardi
CNR-IPCF, Sede Secondaria di Pisa, Largo Pontecorvo 3, 56127 Pisa, Italy

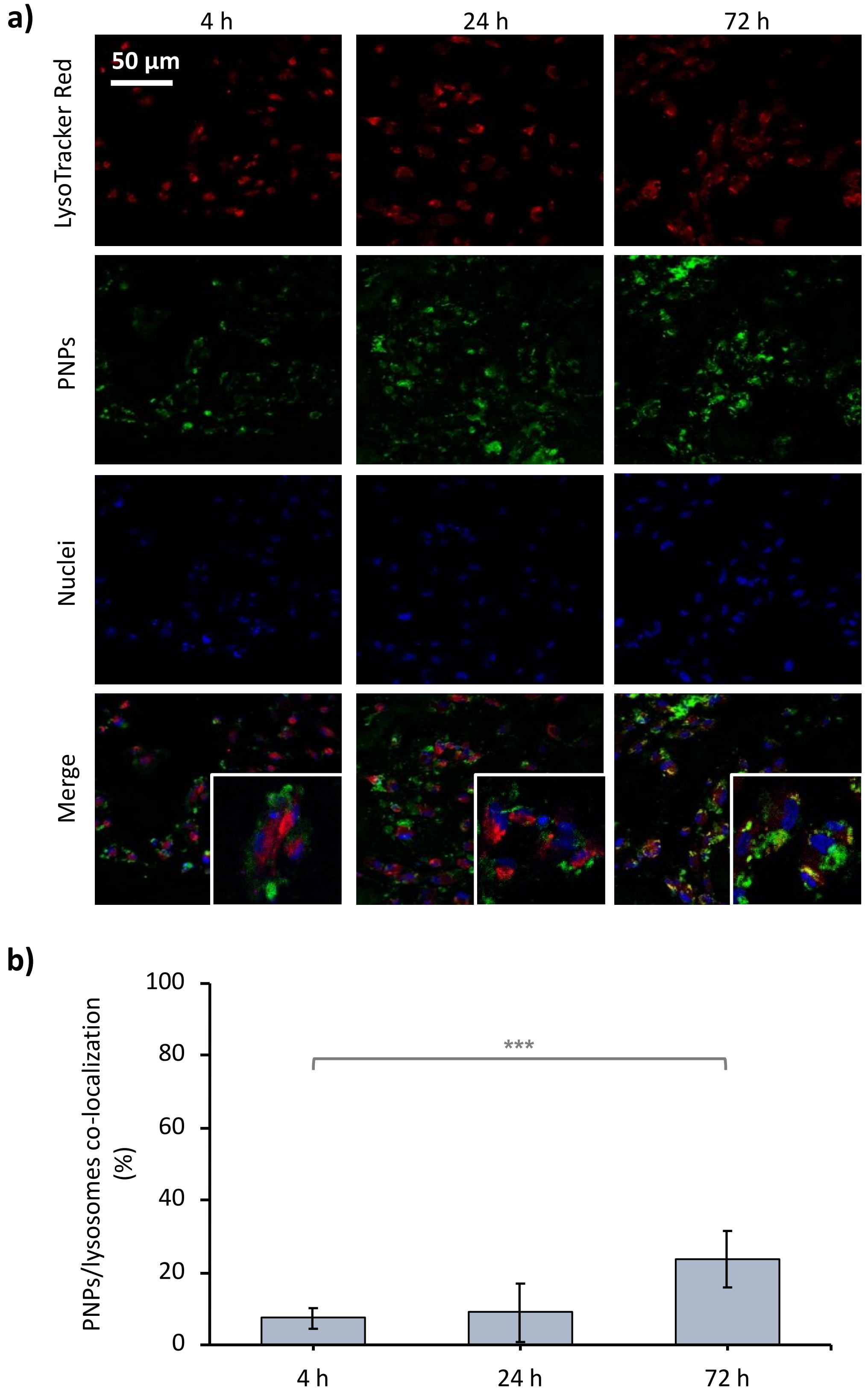

Figure S1. PNPs/lysosomes co-localization. a) Representative confocal images; b) quantitative analysis (*** $p < 0.005$).

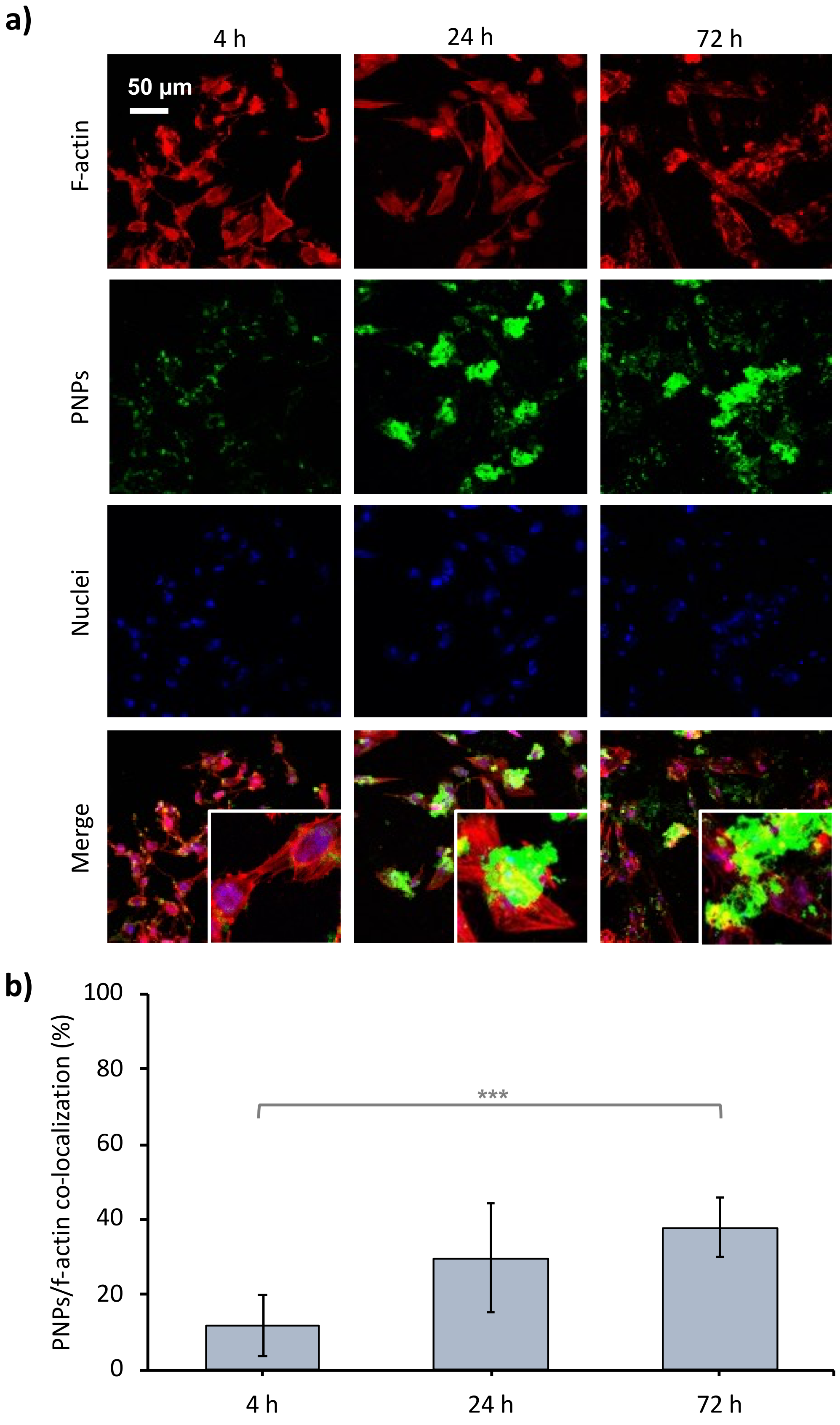

Figure S2. PNPs/f-actin co-localization. a) Representative confocal images; b) quantitative analysis (*** $p < 0.005$).

Table S1. PNP piezoelectric coefficients (d_{33}) of nine PFM measurements on three different nanoparticles.

	***d*₃₃ (pm V⁻¹)**		
	First nanoparticle	Second nanoparticle	Third nanoparticle
First measure	28.0	14.4	18.0
Second measure	23.0	13.5	15.8
Third measure	15.0	13.5	14.0
Mean value ± standard deviation	22.0±5.0	14±0.5	16±1.6
Global mean value ± standard deviation	**17.2 ± 3.5 pm V^{-1}**		

Table S2. List of the primers used for RT-PCR.

**Primer**	**Strand**	**Sequence 5' → 3'**
β-Act	Forward	ctgtgccatccacgaaacta
	Reverse	cgctcaggaggagcaatg
c-Fos	Forward	tactaccactcacccgcaga
	Reverse	cgtgggaatgaagttggcac
c-Jun	Forward	actctttctggctgccttc
	Reverse	acaaacaacactgggcagga
NeuroD1	Forward	acctactaacaacaaaggaaatcg
	Reverse	tccagcttggaggacctt
Lamb1	Forward	gctgccgaaatgacctgt
	Reverse	cccacacttcctctctcct
